# Long-Term Control of Giant Retroperitoneal Liposarcoma by Proton Beam Therapy Combined With Hyperthermia: A Case Report and Literature Review

**DOI:** 10.7759/cureus.103863

**Published:** 2026-02-18

**Authors:** Yuika Suzuki, Masashi Mizumoto, Masafumi Oto, Yukitsugu Kawabata, Hideyuki Sakurai

**Affiliations:** 1 Radiation Oncology, University of Tsukuba Hospital, Tsukuba, JPN; 2 Hematology, Japanese Red Cross Kumamoto Hospital, Kumamoto, JPN; 3 Urology, Asahino General Hospital, Kumamoto, JPN

**Keywords:** hyperthermia, liposarcoma, long-term control, multiple re-irradiation, proton beam therapy (pbt), radiotherapy (rt)

## Abstract

Retroperitoneal liposarcoma is a rare malignancy that often grows to a large size and is difficult to manage because of frequent local recurrence and proximity to critical organs. Surgical resection remains the primary curative treatment, but complete resection is often not feasible in advanced cases.

We report the case of a man in his thirties with a giant retroperitoneal liposarcoma arising near the pancreas. After multiple recurrences and surgical resections over eight years, the tumor progressed to extensive peritoneal dissemination, forming a single massive lesion (maximum diameter 19 cm) that was no longer amenable to surgery. The patient was treated with proton beam therapy (PBT) delivering 70 Gy(relative biological effectiveness (RBE)) in 35 fractions, combined with regional hyperthermia. Because the tumor exceeded the maximum field size of the PBT system, irradiation was performed using a “puzzle-field” technique with multiple stitched fields.

The treated tumor showed marked shrinkage, which was maintained for four years, accompanied by improvement in performance status from 1 to 0. Acute toxicities were mild, and no symptomatic late adverse events were observed. Local control was achieved within the irradiated field, although disease progression later occurred outside the treated area.

This case demonstrates the feasibility of combining proton beam therapy with hyperthermia to achieve durable local control in a giant, unresectable retroperitoneal liposarcoma. The “puzzle-field” irradiation strategy may offer a practical solution for overcoming field size limitations in particle therapy and represents a valuable treatment option in selected advanced cases.

## Introduction

Retroperitoneal tumors are rare, accounting for approximately 0.2% of all malignancies, and retroperitoneal liposarcoma represents one of the most common histological subtypes, comprising 10-20% of cases. Because of their deep anatomical location, these tumors often grow silently and are frequently diagnosed at an advanced size. Surgical resection is generally regarded as the cornerstone of curative treatment for retroperitoneal liposarcoma; however, achieving complete resection is often challenging, particularly in cases of large, recurrent, or multifocal disease. Reported complete resection rates remain low (12-32%), and local recurrence is common even after macroscopically complete surgery [1]. Systemic chemotherapy has demonstrated limited efficacy, and no standard regimen has been established for unresectable or recurrent disease [2]. The role of radiotherapy in retroperitoneal liposarcoma has historically been limited by concerns regarding toxicity to adjacent organs, and published reports have largely focused on adjuvant or perioperative settings [3-5]. More recently, advances in particle therapy, including proton beam therapy (PBT), have enabled improved dose conformality and sparing of surrounding normal tissues, offering a potential treatment option for selected patients with unresectable disease. In addition, hyperthermia has been reported to enhance radiosensitivity and may contribute to improved local tumor control when combined with radiotherapy. Despite these technological advances, the treatment of extremely large retroperitoneal tumors remains technically challenging because of field size limitations inherent to particle therapy systems. In this context, we present a case of a giant retroperitoneal liposarcoma originating near the pancreas that was treated with PBT combined with hyperthermia using a novel “puzzle-field” irradiation technique. This report illustrates the feasibility of this approach in overcoming field size constraints and achieving durable local control in a highly complex clinical situation.

## Case presentation

A man in his thirties was initially diagnosed with pancreatic liposarcoma in June 2019, following tumor resection in his early twenties. Four years later, the disease recurred in the left renal hilum and was surgically resected. Thereafter, the disease recurred approximately every two years, requiring repeated surgical resections. Eight years after the initial operation, widespread peritoneal dissemination developed, rendering further surgical resection infeasible.

The patient was referred for proton beam therapy (PBT) after multiple unsuccessful chemotherapy regimens. At presentation, his Eastern Cooperative Oncology Group performance status was 1, and he complained of abdominal distension and poor appetite. Computed tomography (CT) imaging revealed multiple confluent masses extending from the left retroperitoneum into the abdominal cavity, with the largest lesion measuring 19 × 19 × 29 cm. Because the maximum irradiation field size of our PBT system was limited to 14 cm, a multi-field irradiation approach was required.

The treatment volume was divided into separate fields targeting the upper and lower peritoneal lesions, adrenal lesions, and lesions adjacent to the iliopsoas muscle. Junction regions between adjacent fields were carefully adjusted to avoid excessive dose to organs at risk, particularly the gastrointestinal tract, while minimizing cold spots within the target volume. Dose compensation in the overlap regions was modulated within a range of 80%-120%.

**Figure 1:**
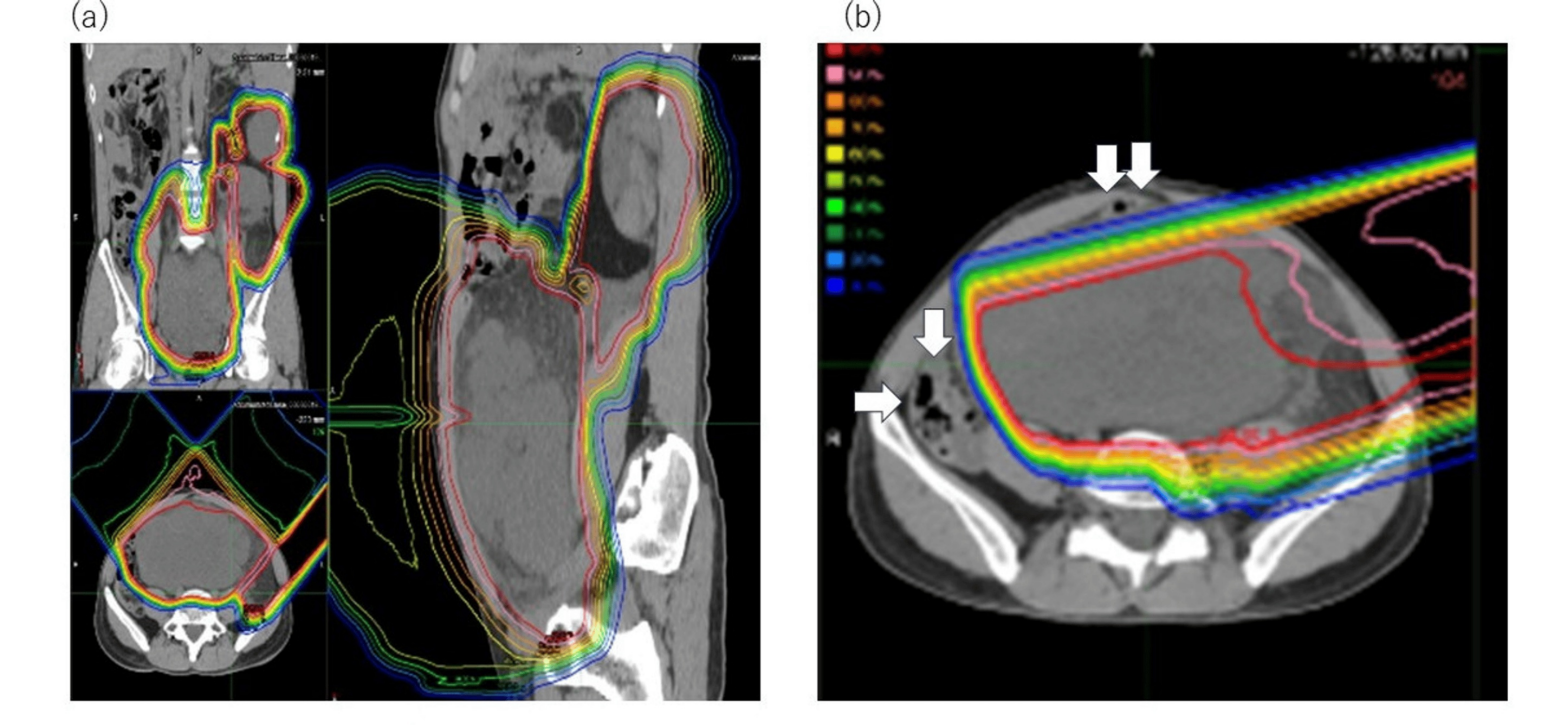
Dose distribution (a) 0–44 Gy(RBE) in 22 fractions. (b) 46–60 Gy(RBE) in 22–30 fractions (using a field that avoids the intestinal tract. The white arrows indicate the gastrointestinal tract).

The patient received PBT to a total dose of 70 Gy(relative biological effectiveness (RBE)) in 35 fractions, delivered concurrently with regional hyperthermia administered twice weekly (60 minutes per session, 1200-1300 W). During the initial 22 fractions (44 Gy(RBE)), treatment planning prioritized tumor coverage with a 5-10 mm margin. From fractions 23-30, dose constraints for the gastrointestinal tract were progressively tightened, and the final treatment phase further minimized radiation exposure to surrounding organs at risk. The dose distribution is shown in Figure 1.

**Figure 2:**
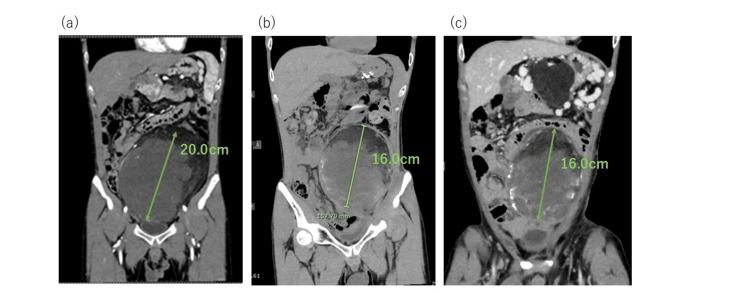
Changes over time (a) Before irradiation. (b) Two years after irradiation; shrinkage of the tumor at the irradiated site is observed. (c) Four years after irradiation; lesions at the irradiated site were not markedly different in size. A recurrent mass is seen in the non-irradiated area.

Acute adverse events were limited to Grade 1 diarrhea and Grade 2 dermatitis. CT imaging performed two years after irradiation demonstrated marked tumor shrinkage, which was maintained four years after treatment. The tumor dimensions decreased from 19 × 19 × 29 cm before treatment to approximately 14 × 10 × 17 cm at two years and remained stable thereafter (Figure 2). Correspondingly, the patient’s performance status improved from 1 to 0, and he reported significant improvement in daily activities and quality of life, including the ability to wear clothing that had previously been restricted by abdominal distension. In contrast, disease progression occurred at non-irradiated sites, including lesions near the liver and in the lung. Re-irradiation with proton beam therapy was performed for newly developed lesions outside the initial irradiation field. The patient continued to receive systemic chemotherapy and hyperthermia for multiple metastatic lesions; however, progressive systemic disease eventually precluded further curative-intent treatment. He was transitioned to palliative care and died 11 months after re-irradiation due to progression of non-irradiated metastatic disease.

## Discussion

Retroperitoneal liposarcomas pose significant treatment challenges due to their size, anatomical location, and tendency for recurrence. While surgical resection remains the cornerstone of treatment, complete resection is often unachievable in cases involving multiple or disseminated lesions [3-5]. Conventional radiotherapy is constrained by the proximity of critical organs such as the gastrointestinal tract. Particle therapy, including proton beam therapy, allows for greater dose conformality and sparing of normal tissue, making it a promising alternative.

Achieving local control in liposarcoma requires high radiation doses, but these are often limited by the risk of severe toxicity, particularly in anatomically complex areas. Conventional photon therapy yields modest outcomes, with five-year local control and survival rates ranging from 29% to 45% and 25% to 35%, respectively [6,7].

Emerging evidence suggests that particle therapy can improve outcomes in inoperable soft tissue sarcomas. A retrospective study involving 57 patients with dedifferentiated liposarcoma treated with particle therapy reported a three-year local control rate of 73.1%, a five-year rate of 67.8%, and a five-year overall survival rate of 62.7% following a median dose of 70.4 Gy in 32 fractions (EQD2 (2 Gy per fraction equivalent dose) = 72.7 Gy). The study also found that gross tumor volume was the sole significant factor for overall survival and that doses exceeding 72.7 Gy EQD2 were associated with better local control [8].

In the present case, multiple peritoneal lesions had fused into a single large tumor closely surrounded by the gastrointestinal tract, which made standard high-dose radiation approaches risky. Therefore, we opted for a slightly extended regimen of 70 Gy(RBE) in 35 fractions to balance efficacy and safety.

A distinctive feature of the present case is that durable local control was safely achieved for several years using a high radiation dose of 70 Gy(RBE) in 35 fractions for a giant tumor measuring up to 19 cm that was closely surrounded by the gastrointestinal tract. In this anatomical setting, dose escalation with conventional photon radiotherapy is often precluded by the tolerance of adjacent organs at risk, particularly the gastrointestinal tract, for which doses exceeding approximately 50 Gy are associated with a high risk of severe toxicity, as well as the kidneys, where the irradiated volume receiving more than 15 Gy must be kept to a minimum. Even with proton beam therapy, standard irradiation approaches are challenged by the extreme tumor size, which exceeded the maximum field size of the delivery system and would ordinarily preclude safe high-dose treatment. In the present case, these limitations were addressed by employing a multi-field “puzzle-field” irradiation strategy with careful dose modulation at field junctions, allowing adequate tumor coverage while maintaining organ-at-risk constraints. This technical adaptation enabled safe delivery of a curative-intent dose of proton beam therapy in an otherwise prohibitive clinical scenario and likely contributed to the durable local control observed over several years.

The combination of radiotherapy with hyperthermia has also been shown to enhance radiosensitivity by interfering with intracellular protein structures and DNA repair pathways [9,10]. In our patient, hyperthermia was applied concurrently with proton beam therapy, particularly in regions where full radiation dosing was limited, such as field junctions and areas near sensitive organs. Hyperthermia therapy was administered twice weekly for a total of 17 sessions. Starting at a maximum output of 1300 W, the patient was able to reach 1500 W by the final day. Although the specific contribution of hyperthermia cannot be isolated in a single case, this combined approach may have supported sustained tumor shrinkage and symptom relief. While the ultimate outcome was affected by disease progression outside the irradiated field, this case illustrates the feasibility of combining proton beam therapy and hyperthermia to achieve local control in a highly selected and complex clinical setting.

## Conclusions

This case suggests that proton beam therapy combined with hyperthermia may be feasible for achieving durable local control in selected patients with unresectable retroperitoneal liposarcoma. The “puzzle-field” technique may represent a practical approach to overcoming technical field size limitations in particle therapy.
